# Effect of Silk Fibroin Biomaterial Coating on Cell Viability and Intestinal Adhesion of Probiotic Bacteria

**DOI:** 10.4014/jmb.2103.03031

**Published:** 2021-04-05

**Authors:** Gicheol Kwon, Bohye Heo, Mi Jin Kwon, Insu Kim, Jaeryang Chu, Byung-Yong Kim, Byoung-Kook Kim, Sung Sun Park

**Affiliations:** 1 R&D Center, Chong Kun Dang Healthcare, Seoul 07249, Republic of Korea; 2 Probiotics Research Laboratory, Chong Kun Dang Bio Research Institute, Ansan 15604, Republic of Korea

**Keywords:** Probiotics, silk fibroin coating, freeze drying, intestinal cell adhesion

## Abstract

Probiotics can be processed into a powder, tablet, or capsule form for easy intake. They are exposed to frequent stresses not only during complex processing steps, but also in the human body after intake. For this reason, various coating agents that promote probiotic bacterial stability in the intestinal environment have been developed. Silk fibroin (SF) is a material used in a variety of fields from drug delivery systems to enzyme immobilization and has potential as a coating agent for probiotics. In this study, we investigated this potential by coating probiotic strains with 0.1% or 1% water-soluble calcium (WSC), 1% SF, and 10% trehalose. Under simulated gastrointestinal conditions, cell viability, cell surface hydrophobicity, and cell adhesion to intestinal epithelial cells were then measured. The survival ratio after freeze-drying was highest upon addition of 0.1% WSC. The probiotic bacteria coated with SF showed improved survival by more than 10.0% under simulated gastric conditions and 4.8% under simulated intestinal conditions. Moreover, the cell adhesion to intestinal epithelial cells was elevated by 1.0-36.0%. Our results indicate that SF has positive effects on enhancing the survival and adhesion capacity of bacterial strains under environmental stresses, thus demonstrating its potential as a suitable coating agent to stabilize probiotics throughout processing, packaging, storage and consumption.

## Introduction

Probiotics are live microorganisms that provide health benefits in the human body through an adequate intake [1, 2]. Various effects have been reported, such as reduction in diarrhea, inhibition of pathogenic bacteria, lowered blood cholesterol, and improvements in liver cirrhosis and obesity [3-6]. Thanks to these effects, probiotics have been widely applied to food products, drugs and feed additives, with their use increasing each year globally [7, 8]. The most well-known genera of probiotics include *Lactobacillus*, *Bifidobacterium*, *Streptococcus*, *Leuconostoc*, *Pediococcus*, *Enterococcus*, and *Lactococcus* [3, 4]. In general, the commercialization of a probiotic product involves the cultivation of microorganisms and the processes of concentration and drying or freeze-drying for subsequent processing into a powder, granular, tablet, or capsule-type product [9]. This processing requires steps such as large-scale cell culture, collection of microbial cells, freeze-drying and pulverizing, in which microorganisms are exposed to physical or chemical stresses [10]. For example, cells are under the influence of osmotic pressure due to the process of concentration during the collection of microbes. While during freeze-drying, they are also under the influence of both temperature and osmotic pressure due to ice crystallization and dehydration [10]. In the steps of pulverizing and packaging, exposure to high temperatures and pressure decreases the viability of microbial cells as their membrane lipids are oxidized [10]. Microbial cells face various stress conditions, even after intake via the human gastrointestinal tract [11]; they are exposed to a strongly acidic (~pH2) environment in the stomach, and in the small intestine, various digestive enzymes and bile salt exert an influence [10]. Even if the cells survive to reach the large intestine, various hazardous compounds and reactive oxygen species further inhibit their growth. Microbial cells also need to adhere to intestinal epithelial cells amidst competition with other gut microbiota [10]. Thus, various materials and techniques have been developed to enable these beneficial microorganisms to survive and settle among the other gut microbiota with stability [12]. Of note is the development of various coating techniques for probiotics. Initially, capsule-type intestinal coating agents and micro-capsules using gelatin, sugars or gums were applied [11]. Subsequently, methods such as double-coating using proteins and polysaccharides, triple-coating with additional nanoparticles, and four-layer coatings with the addition of water-soluble polymers, hyaluronic acid, or porous coating agents, have been developed [11, 13-15]. A number of drawbacks, however, include contamination during complex processing steps, the added cost of processing, and the use of expensive coating agents, which together reduce probiotic economic feasibility and commercialization [16, 17]. Another drawback is that such coatings prevent direct contact between probiotic bacteria and intestinal cells to the extent that their effects may not be manifested at an adequate level [18].

The intestinal adhesion ability of probiotic bacteria is an important criterion in selecting candidate strains due to following reasons. First, the adherence of probiotic bacteria to mucus layer prolong the retention time of microorganism in intestinal tract. Second, the residence time of probiotic bacteria in the intestine influence the number of beneficial metabolites, namely postbiotics such as short-chain fatty acids (SCFA), vitamins, and peptides [19, 20]. In addition, as receptors on the intestinal epithelial cells are dominated by probiotics, the host can be protected against pathogenic infections [20]. The first step in the intestinal adhesion of probiotics is physical binding and involving hydrophobic interactions, between the surface of probiotic bacteria and intestinal epithelial cells [20]. It is thus probable that the surface hydrophobicity of probiotic bacteria has an influence on binding to intestinal epithelial cells [21]. Therefore, adhesion between probiotic bacteria and intestinal epithelial cells can be predicted based on probiotic cell surface hydrophobicity [21]. However, there is a general lack of studies regarding coating materials for probiotics in terms of their influence on intestinal adhesion ability and cell surface hydrophobicity.

Silk fibroin (SF) is 70-80% of the natural protein polymers constituting the silk obtained from *Bombyx mori* cocoon, which protects pupae against adverse environments. It is a long and thin fibrous protein produced by the degumming of silk [22, 23]. As it is non-toxic, while showing a high level of biocompatibility and allowing easy processing and ready supply via silkworm farming, SF has been widely used in drug delivery, surgical sutures, vascular tissue regeneration, and enzyme immobilization [23]. The key amino acids in SF are glycine (~43%) and alanine (~30%) known as essential amino acids which ensure strong hydrophobicity [23]. The β-sheet structure of SF contains hydrogen bonds and offers hydrophobic interactions to provide a robust network [24]. Thus, SF has been investigated in numerous previous studies regarding its efficacy such as anti-inflammatory activity, wound healing effects, strong cell adhesion ability, and outstanding adhesion to mucin [25, 26]. SF with the robust networks, strong hydrophobicity, and excellent cell adhesion ability can protect probiotic bacteria from adverse environments such as gastric acid and bile acid, and have the potential to contribute to improving intestinal adhesion. However, no study has yet investigated the application of SF as a coating agent for enhancing probiotic stability and intestinal adhesion ability.

**Fig. 1:**
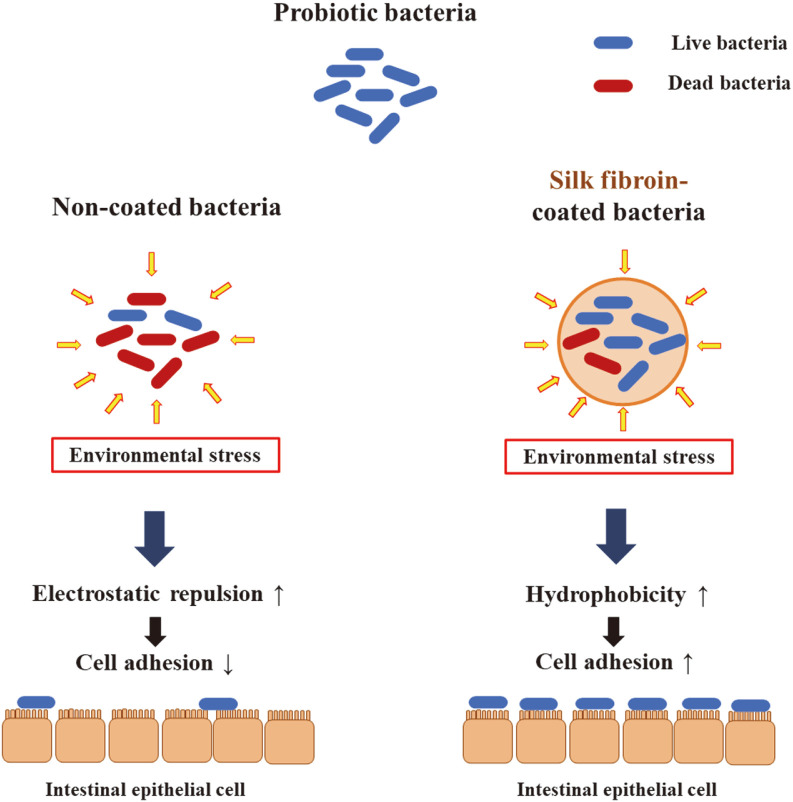
The schematic illustration of silk fibroin coating effects on cell surface hydrophobicity, and intestinal adhesion ability.

The purpose of this study was to apply SF coating to bacterial strains isolated from foods and infant gut microbiota, and to evaluate acid and bile tolerance, cell surface hydrophobicity, and intestinal adhesion ability. First, to determine optimum coating conditions, changes in the concentration of water-soluble calcium (WSC) were examined and the survival ratio after freeze-drying was measured. In addition, bacterial strains without coatings, microbes with SF coating, and ethanol-treated silk fibroin (ETSF) coating were compared in terms of acid and bile tolerance, cell surface hydrophobicity, and intestinal adhesion ability (Fig. 1). The results collectively indicated that SF coating ensured a reliable coating for enhancing cell adhesion ability, and the stable adhesion of bacterial strains to intestinal epithelial cells has been verified through immunofluorescence staining.

## Materials and Methods

### Bacterial Strains

The bacterial strains used in this study were: *Enterococcus faecium* KCTC 13115BP (EF-3), *Streptococcus thermophilus* KCTC 14471BP (ST-27), *Bifidobacterium animalis* subsp. *lactis* KCTC 13116BP (BL-5), *Bifidobacterium bifidum* KCTC 13114BP (BB-1), and *Lactobacillus acidophilus* KCTC 13117BP (LA-7). The strains were isolated from fermented foods and newborn infant feces, and deposited at Korean Collection for Type Cultures (KCTC). The isolation was performed according to the Coeuret’s method by using selective media [27]. For the cultivation and maintenance of these strains, blood-liver (BL) medium (BD Difco, USA) was used for the strains of the *Bifidobacterium* strains, and for all other strains, deMan-Rogosa-Sharpe (MRS) medium (BD Difco) was used.

### Identification Using 16S rRNA Gene Sequence Analysis

For the identification of the strains, the 16S rRNA gene was amplified and sequenced. The genomic DNA was extracted according to the instruction provided by the manufacturer of DNA extraction kit (Bioneer, Korea). The 16S rRNA gene was amplified using the universal bacterial primer set: 27F 5’ (AGA GTT TGA TCM TGG CTCAG) 3’ and 1492R 5’ (TAC GGY TAC CTT GTT ACG ACT T) 3’ [28]. BLAST analysis for 16S rRNA gene sequences was done on the EzBioCloud database (https://www.ezbiocloud.net/).

### Preparation of Silk Fibroin Coating Materials

In this study, SF obtained from silk cocoons after degumming was used (Worldway Co., Korea). To increase solubility and the rate of absorption in the body, the SF was treated with 5% Protamax (Bision Corporation, Korea) and T100 (Bision Corporation) at 60-80°C for 6 - 10 h. Enzymes used in the pretreatment of SF were inactivated at 95°C for 2 h. The pretreated SF was treated with ethanol following Nogueira’s method to produce an elaborate structure [29]. To enhance the coating ability for bacterial strains, the enzyme-treated SF was treated with 30%ethyl alcohol at 25°C for 18-24 h, to produce ethanol-treated silk fibroin (ETSF).

### Treatment of Water-Soluble Calcium and Silk Fibroin, and Production of SF-Coated Probiotic Powder

Each bacterial strain was cultured in 5 L lab-scale fermenters (Biocns Co., Korea). 0.1% SF-coating material was added to the BL medium for the *Bifidobacterium* strains and MRS medium for all other strains. The culture temperature and stirring speed were 37°C, 120 rpm, and N_2_ gas was used for substitution to create an anaerobic environment. Every two hours, N_2_ gas consumption rate was estimated and OD_600 nm_ was measured using an 800 TS Absorbance Reader (BioTek Inc., USA) so as to collect the cells in stationary phase.

To produce SF-coated probiotic powder, the bacterial liquid culture in stationary growth phase was enriched using a high speed centrifuge Supra R12 (Hanil Co., Korea) at 11,500 ×*g*, 4°C for 20 min. First, by varying the concentration of WSC to 0%, 0.1%, and 1% for the enriched culture solution, an optimal concentration was determined. 0.1% WSC was added to the probiotic powder for cell surface hydrophobicity and intestinal epithelial cell experiments. Sodium phosphate buffer (pH 6.8) was used as a base buffer to reach the concentration shown in Table 1 [30]. Finally, the probiotic powder was obtained through freeze-drying using a freezone 12 L-freeze dryer (Labconco Co., USA).

### Acid and Bile Tolerance Test under Simulated Gastrointestinal Conditions

To verify the effects of SF as a coating material, acid and bile tolerances were estimated using a modified version of Hansen’s method [31]. 10% freeze-dried probiotic powder was added to simulated gastric fluid or intestinal fluid, and the mixture was stirred at 100 rpm/min for 2 h. To produce the simulated gastric or intestinal fluid, HCl and NaOH were used for adjustment to pH 2.5 and pH 7.0, respectively. The simulated intestinal fluid was mixed with 0.5% oxgall (BD Biosciences, USA). After treatment, bacterial viability in the solution was measured using the plate count method [32]. The diluted solution was poured onto MRS or BL agar for 48 h culture at 37°C. Plates with 30-300 colonies were counted.

### Cell Surface Hydrophobicity Analysis

To indirectly monitor the intestinal adhesion of bacterial strains, the cell surface hydrophobicity was examined using a slightly modified version of Krausova’s method [33]. Phosphate buffered saline (PBS) was used to dilute the probiotic powder with or without SF to OD_600 nm_ = 0.5. The diluted samples were mixed with toluene at 37°C and reacted for 20 mins. At the end of the reaction, toluene was removed and OD_600 nm_ of the remaining solution was measured for the following equation:

$$\frac{\left(H_{0})-(H_{t}\right)}{\left(H_{0}\right)}\times 100=\mathrm{Surface}\mathrm{hydrophobicity}(\%)$$

where t: time (min), *H*_0_: Optical density of sample at initial, *H*_t_: Optical density of sample after t times [33].

### Cell Adhesion Assay and Immunofluorescence Staining of Bacterial Strain

Human colorectal adenocarcinoma cell line HT-29 was used to test the adhesive abilities of bacterial strain. For the cultivation and maintenance of the cell line, a standard procedure was followed [34]. The cells were cultured in 5% CO_2_ in a humidified incubator at 37°C, using Dulbecco’s modified Eagle’s medium (DMEM) with 10% fetal calf serum (FCS) (Sigma-Aldrich, Germany) and 100 U/ml penicillin - 100 μg/ml streptomycin (Thermo Fisher Scientific, USA). HT-29 cells that had formed a monolayer on the plate were treated with 0.5% trypsin-EDTA for 2 min to detach the cells. 1 × 10^5^ cells were then aliquoted into a 12-well plate. Adhesion assays were carried out using a modified version of Hirono’s method [35]. HT-29 cells were washed three times with PBS, and DMEM without antibiotics was used to replace the medium. The probiotic powder was diluted to 1 × 10^9^ CFU/ml with PBS buffer and 100 μl was aliquoted to each well on the plate. The cells were cultured at 37°C in a 5% CO_2_ humidified incubator for 2 h. At the end of the reaction, any non-adherent cells were removed by washing five times with PBS buffer, and HT-29 cells and bacterial strain were separated through 2 min of treatment with 0.5% trypsin-EDTA. Following serial dilution of the isolated bacterial strain, MRS or BL agar plates were used for 48 h cultivation at 37°C to count the number of viable cells according to the plate count method [32].

To confirm bacterial adhesion of strain ST-27 to HT-29 cells, immunofluorescent staining assay was followed according to Alemka’s method [36]. Strain ST-27 were diluted and inoculated into HT-29 cells grown on 8-well cell culture slides (SPL, Korea). The cells were cultured in a 37°C, 5% CO_2_ humidified incubator for 2 h [34]. The cells were fixed in 4% formaldehyde for 30 min, then treated for 15 min with 0.1% Triton X-100 (Sigma-Aldrich) and 3% bovine serum albumin (BSA) (Sigma-Aldrich) for permeabilization and membrane blocking. The anti-peptidoglycan antibody 3F6B3 (10H6) (Bio-Rad, USA) and goat anti-mouse IgG H&L Alexa Fluor 488 (Abcam, USA) were added to reaction. For immunofluorescent imaging, mounting medium with DAPI (Abcam) was used, and observations were conducted using a confocal microscope LSM 700 (Zeiss, Germany).

### Statistical Analysis

All data are expressed as means ± SD. For data analysis, one-way analysis of variance (ANOVA) was used. For multiple comparisons of significant differences, Dunnett’s test was performed using GraphPad Prism software (GraphPad Software, USA) [37]. The level of statistical significance was set as *p < 0.05, **p < 0.01, ***p < 0.001.

## Results and Discussion

### Identification of Bacterial Strains

As a result of 16S rRNA gene sequence analysis and comparison with the type strain of each species, EF-3 was identified to *Enterococcus faecium* (99.7% similarity), ST-27 was *S. thermophilus* (99.9%), BL-5 was *Bifidobacterium animalis* ssp. *lactis* (100%), BB-1 was *B. bifidum* (99.8%), and LA-7 was *L. acidophilus* (100%). These results were summarized in Table 2.

### Effects of Water-Soluble Calcium on Survival Ratio after Freeze Drying Process

**Fig. 2:**
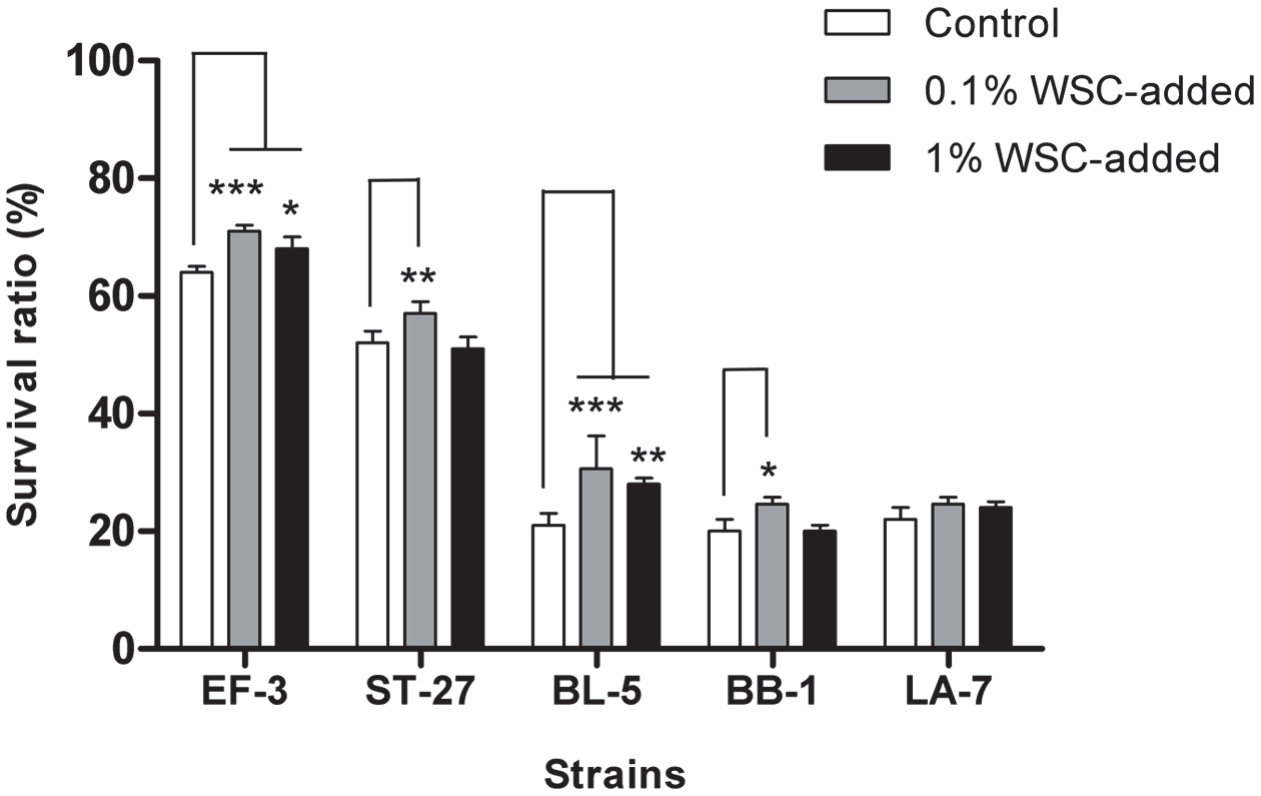
Survival ratio of the probiotic bacteria depending on the different concentration of water-soluble calcium concentration during the freeze drying process. Bar charts show the mean ± standard error of the mean. Statistical significance was determined using one-way ANOVA with Dunnett’s multiple comparisons test. *p < 0.05, **p < 0.01, ***p < 0.001 compared to the control group.

The protective effects of WSC during microbial cultivation with silk fibroin (SF) were evaluated. The effects were analyzed based on the survival ratio of bacterial strain upon freeze-drying with the addition of 0%, 0.1%, and 1% of WSC. The process without WSC led to the survival ratio of 64.0%, 51.7%, 22.0%, 21.3%, and 19.7% of EF-3, ST-27, LA-7, BL-5, and BB-1, respectively (Fig. 2). The survival ratio upon addition of 0.1% WSC was 71.0%, 57.0%, 24.7%, 30.7%, and 24.7%, all of which exhibited an improvement in survival (Fig. 2). Notably, EF-3, ST-27, BL-5, and BB-1 showed a substantial improvement in survival. In contrast, the addition of 1% WSC did not show a significant difference in survival to the addition of 0.1% across all strains, compared to the control (Fig. 2). In the freeze-drying process, the cell membrane may be damaged due to osmotic pressure on the cell surface as water evaporates and by crystallization of intracellular water. Here, WSC could protect cell membranes against osmotic pressure by interacting with proteins [38, 39]. Ainsley *et al*. also reported that interactions between WSC and whey protein increase the survival of *Lactobacillus rhamnosus* upon freeze-drying [38]. The lack of increase in the survival of bacterial strain upon freeze-drying as the concentration of WSC increased from 0.1% to 1% is presumed to be due to the high concentration of calcium ions being unable to defend the cell membrane against SF crystals as effectively. While 0.1% WSC leads to stable membrane formation on the surface of bacterial strain to protect the cells against osmotic pressure and crystallization, 1% WSC could cause excessive crystal formation on the surface of bacterial strain. According to Zheng *et al*., a high concentration of salt ions during freeze-drying causes physical damage at the cell surface via an excess crystal formation [39]. Another study indicated that a low concentration of calcium ions enabled SF to acquire a stable β-sheet structure, and the membrane thus formed was robust [40]. LA-7, compared to the other strains, was not influenced by WSC. This may be an inherent property of the LA-7 strain, which implies relatively low interactions with calcium ions at the cell surface. In fact, Pech-Canul *et al*. reported that the coating effect was under the influence of not only the coating material and methods, but also the strain of the probiotic bacteria involved [41]. To conclude, the addition of 0.1% WSC led to the highest stability of coating efficiency for probiotic bacteria survival after freeze-drying.

### Protective Effects of Silk Fibroin Coating in Gastrointestinal Fluid

To examine the protective effects of SF under simulated gastrointestinal conditions, each bacterial strain was treated with simulated intestinal fluid and simulated gastric fluid. The effect of ethanol-treated SF (ETSF) coating was also evaluated. The SF coatings of bacterial strain were shown to improve the overall survival ratio across all strains (Table 3). The survival ratio increased by 13.3-31.3% in simulated gastric fluid and by 4.8-23.5% in simulated intestinal fluid. The survival ratio was highest for EF-3, followed by BL-5, BB-1, LA-7, ST-27 in simulated gastric fluid, and for LA-7, followed by BL-5, BB-1, EF-3, and ST-27 in simulated intestinal fluid. The ETSF coatings were shown to improve the survival ratio of bacterial strain by 20.7-47.9% and 16.2-25.8% in simulated gastric fluid and simulated intestinal fluid, respectively, compared to the control (Table 3).

SF is enriched with β-sheet structures in an anti-parallel arrangement to create a robust network [24]. Moreover, the interaction of SF with calcium ions is known to create a firm membrane gel [40]. The resulting β-sheet network implies a defense against acid and bile stress on the cell surface. As a result, the SF membrane on the probiotic cell surface could protect the cell against external stress and increase the survival of bacterial strain. Particularly, when SF was treated with ethanol, the protective effect of SF is further enhanced. It has already been shown in previous studies that ethanol treatment of SF increases the β-sheet structure content and creates a stronger film [23, 29]. To conclude, coating with SF was shown to enhance acid and bile tolerance, increasing the survival ratio of probiotic bacteria.

### Changes to Cell Surface Hydrophobicity by Silk Fibroin Coating

**Fig. 3:**
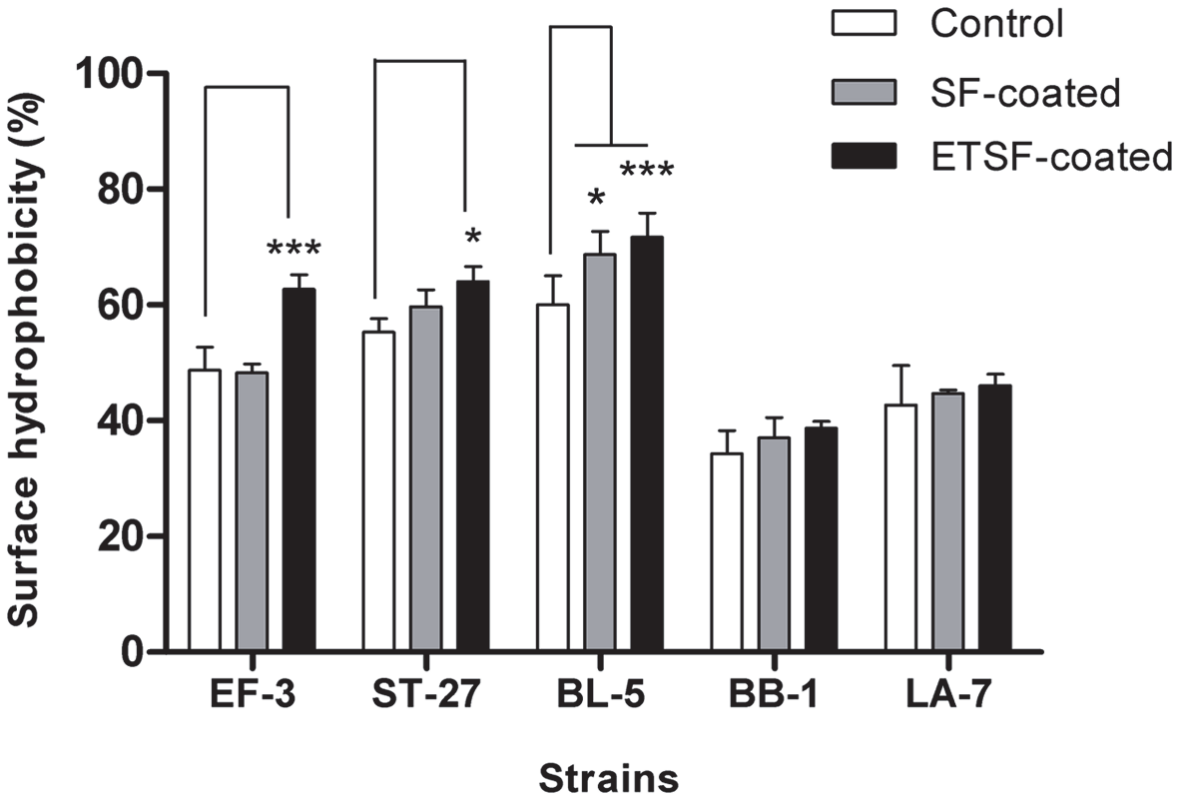
Cell surface hydrophobicity of the probiotic bacteria coated with silk fibroin. Bar charts show the mean ± standard error of the mean. Statistical significance was determined using one-way ANOVA with Dunnett’s multiple comparisons test. *p < 0.05, **p < 0.01, ***p < 0.001 compared to the control group.

To examine changes in cell surface properties caused by SF coating, cell surface hydrophobicity was evaluated. The surface hydrophobicity of EF-3, ST-27, BL-5, BB-1, and LA-7 without SF coating was 48.7%, 55.3%, 60.0%, 34.3%, and 42.7%, respectively (Fig. 3). The surface hydrophobicity generally increased across all strains upon SF coating, and a notable, significant, increase in hydrophobicity to 68.7% was shown by BL-5. The surface hydrophobicity upon SF coating showed even greater increases to 62.7%, 64.0%, and 71.7% for EF-3, ST-27, and BL-5, respectively (Fig. 3). Meanwhile, the surface hydrophobicity generally increased for BB-1 and LA-7 based on SF coating, but without statistical significance. Therefore, the use of SF as a coating material is expected to significantly improve the surface hydrophobicity of probiotic bacteria.

Physicochemical changes in coating materials or processes employed could induce substantial differences in the properties and efficiency of the final product [41]. SF contains glycine and alanine as key amino acids which also exhibit hydrophobicity such that the cell surface hydrophobicity is predicted to increase after SF coating. In fact, Zhang and Dai showed a marked improvement in hydrophobicity upon ethanol treatment of SF nanofibers [42]. The lack of a significant difference in hydrophobicity for BB-1 and LA-7 is presumed to be due to strain specificity, as observed in a previous experiment. The variation of the coating effect according to the bacterial strain may be attributed to the specific coating mechanism or experimental conditions, for which further studies should be conducted.

### Adhesion Ability to Intestinal Epithelial Cells

**Fig. 4:**
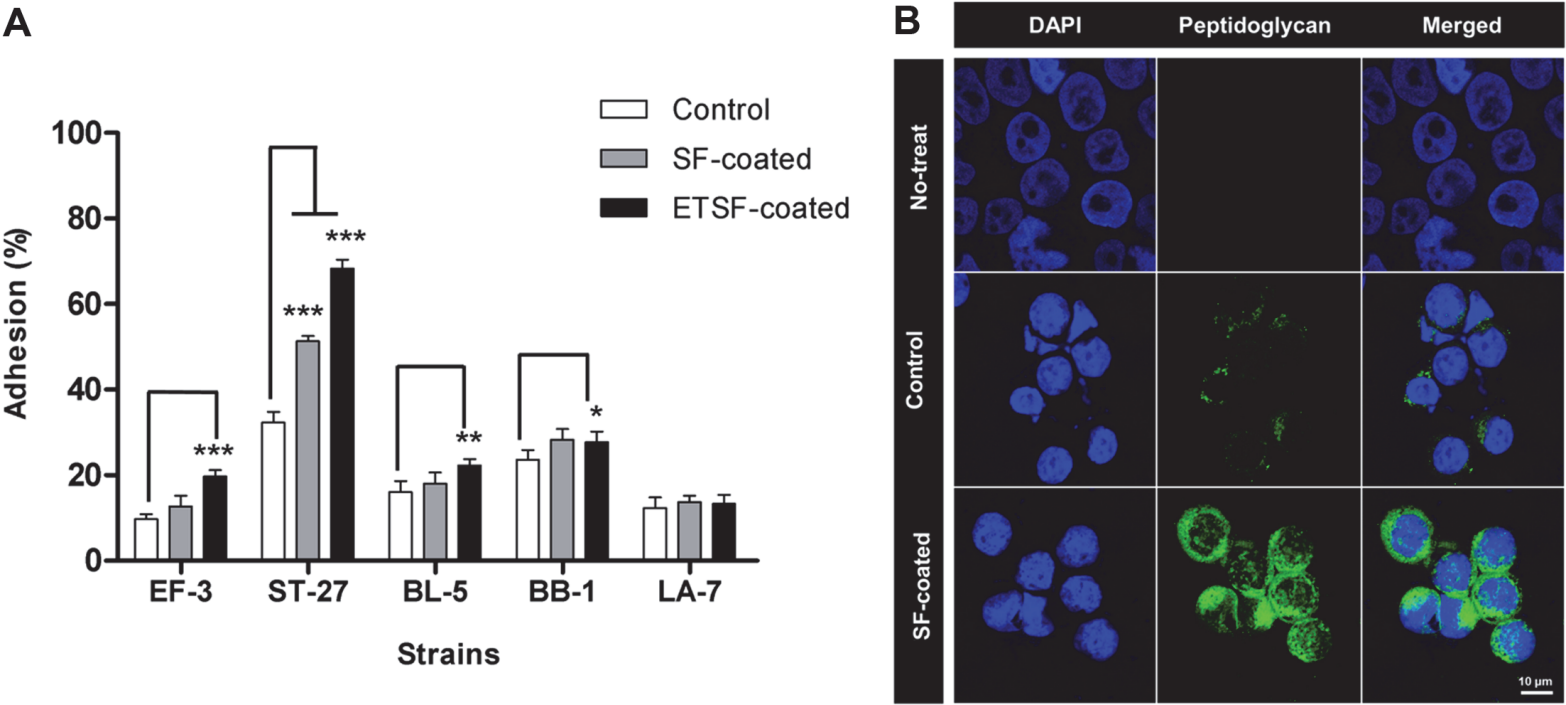
Cell adhesion and immunostaining image of the probiotic bacteria coated with silk fibroin. **A**) Bar charts show the mean ± standard error of the mean. Statistical significance was determined using one-way ANOVA with Dunnett’s multiple comparisons test. *p < 0.05, **p < 0.01, ***p < 0.001 compared to the control group. **B**) HT-29 cell was stained with DAPI (blue) and ST-27 with or without coating silk fibroin was stained with anti-peptidoglycan antibody as primary antibody and goat anti-mouse Alexa fluor 488 (green). Magnification, 630×. Scale bars, 10 μm.

HT-29 cells are intestinal epithelial cells that produce mucin, which is widely used in in vitro tests of intestinal adhesion abilities of bacterial strain [43]. In the control group, the intestinal adhesion ability of EF-3, ST-27, BL-5, BB-1, and LA-7 was 9.7%, 32.3%, 16.0%, 23.6%, and 12.3%, respectively (Fig. 4A). In the group with SF coating, the intestinal adhesion ability was shown to have increased to 12.7%, 51.3%, 18.0%, 28.3%, and 13.7% for EF-3, ST-27, BL-5, BB-1, and LA-7, respectively (Fig. 4A). While the adhesion ability increased for EF-3, BL-5, BB-1, and LA-7, no statistical significance was found. In the group with ETSF coating, the adhesion ability was 19.7%, 68.3%, 22.3%, 27.7%, and 13.3% for EF-3, ST-27, BL-5, BB-1, and LA-7, respectively, showing an improvement compared to the control (Fig. 4A). This difference was statistically significant for all strains except LA-7. A particularly large increase was shown by EF-3, ST-27, and BL-5. Meanwhile, LA-7 showed an increase in adhesion ability in general, based on the SF coating, but no statistical significance was found.

These results were indicative of a similar pattern involving cell surface hydrophobicity and intestinal adhesion ability, suggesting that the enhanced hydrophobicity is correlated with improved adhesion ability of bacterial strain (Figs. 3 and 4A). The mucin in the intestinal cells comprises glycoproteins with negative charges, so that contact between mucin and non-hydrophobic probiotic bacteria could cause mutual repulsion, thereby inhibiting intestinal adhesion [44, 45]. Thus, a surface coating of probiotic bacteria that increases hydrophobicity would decrease repulsion toward mucin and increase the intestinal adhesion ability of the bacteria [44]. This was in accord with Duary *et al*., where *Lactobacillus* strains with high surface hydrophobicity exhibit strong adhesion capacity for intestinal epithelial cells [46]. In addition, a larger number of probiotic bacteria could be aided in adhesion to intestinal epithelial cells, as high surface hydrophobicity increases intercellular auto-aggregation [47].

Among the bacterial strains used in this study, ST-27 exhibited the strongest intestinal adhesion ability (Fig. 4A). According to Grigoryan *et al*., *Streptococcus thermophilus* exhibits a high level of auto-aggregation, which may account for the highest adhesion ability shown by ST-27 [48]. The ST-27 was shown to be adherent to the surface of HT-29 cells (Fig. 4B). Anti-peptidoglycan antibody was used to stain ST-27, and through immunofluorescent imaging, control and SF-coated samples were compared. Both the control and SF-coated ST-27 were found to have adhered to the surface of HT-29 cells (Fig. 4B). In addition, compared to the control, a greater number of bacterial strain in the SF-coated group were found to have adhered to the surface of HT-29 cells (Fig. 4A). The SF-coated group also showed a high level of auto-aggregation on the surface of HT-29 cells. This is also presumed to be a factor contributing to the greater intestinal adhesive capacity.

The findings in this study indicated that the addition of WSC and SF coating led to enhanced survival of bacterial strain after freeze-drying, as well as higher levels of cell surface hydrophobicity and intestinal adhesion ability. The survival ratio after freeze-drying was highest when 0.1% WSC was added, while the SF coating was shown to have enhanced the acid and bile tolerance as well as cell surface hydrophobicity and adhesion to intestinal epithelial cells. In addition, ethanol treatment was shown to have further augmented the positive effects of SF coating. These results suggested that the SF material, being already widely used in various fields, could be used as an effective coating agent for probiotics to ensure their stable arrival in the intestine with enhanced adhesion ability.

## Figures and Tables

**Table 1:** Composition of cell mixture for probiotic powder production.

Materials	Concentration (%)		

	Control	SF-coated	ETSF-coated
SF^a^	-	1	-
ETSF^b^	-	-	1
WSC^c^	0.1	0.1	0.1
Trehalose	20	20	20

^a^SF : Silk fibroin.

^b^ETSF : Ethanol-treated silk fibroin.

^c^WSC : Water-soluble calcium.

**Table 2:** Species identification of the bacterial strains.

Strain	Species identification	Identity (%)	Strain No.	Origin
EF-3	*Enterococcus faecium*	99.73	KCTC 13115BP	Fermented fruits
ST-27	*Streptococcus thermophilus*	99.93	KCTC 14471BP	Raw milk
BL-5	*Bifidobacterium animalis* ssp. *lactis*	100.00	KCTC 13116BP	Newborn infant
BB-1	*Bifidobacterium bifidum*	99.79	KCTC 13114BP	Newborn infant
LA-7	*Lactobacillus acidophilus*	100.00	KCTC 13117BP	Newborn infant

**Table 3:** The bile salt and acid tolerance of the probiotic bacteria coated with silk fibroin.

Strain	Conditions	Survial ratio (%)		

		Control	SF-coated	ETSF-coated
EF-3	SGF^a^	48.37±5.91	61.63±1.98	71.93±1.47
	SIF^b^	40.27±2.64	63.77±2.89	65.67±7.02
ST-27	SGF^a^	24.33±2.39	55.63±2.30	72.20±7.86
	SIF^b^	32.17±2.84	37.00±4.36	48.33±3.06
BL-5	SGF^a^	42.93±4.18	52.90±2.33	72.03±1.27
	SIF^b^	47.50±3.12	63.17±3.55	73.33±2.08
BB-1	SGF^a^	42.70±5.07	57.80±2.36	63.40±3.54
	SIF^b^	44.00±4.00	56.67±5.86	61.33±4.16
LA-7	SGF^a^	34.40±2.13	63.13±3.00	74.47±3.40
	SIF^b^	58.00±2.65	63.00±2.65	76.67±1.53

The survival ratio are expressed as mean±standard deviation of the measurements of three biological replicates.

^a^SGF : Simulated gastric fluid.

^b^SIF : Simulated intestinal fluid.
